# The Effects of Maternal Nutrient Restriction during Mid to Late Gestation with Realimentation on Fetal Metabolic Profiles in the Liver, Skeletal Muscle, and Blood in Sheep

**DOI:** 10.3390/metabo14090465

**Published:** 2024-08-23

**Authors:** Brandon I. Smith, Manuel A. Vásquez-Hidalgo, Xiaomeng Li, Kimberly A. Vonnahme, Anna T. Grazul-Bilska, Kendall C. Swanson, Timothy E. Moore, Sarah A. Reed, Kristen E. Govoni

**Affiliations:** 1 Department of Animal Science, University of Connecticut, Storrs, CT 06269, USAsarah.reed@uconn.edu (S.A.R.); 2 Department of Animal Sciences, North Dakota State University, Fargo, ND 58102, USA; manuel.vasquezhidalgo@sdstate.edu (M.A.V.-H.); anna.grazulbilska@ndus.edu (A.T.G.-B.); kendall.swanson@ndsu.edu (K.C.S.); 3 Department of Statistics, University of Connecticut, Storrs, CT 06269, USAtimothy.e.moore@uconn.edu (T.E.M.)

**Keywords:** circulation, developmental programming, liver, maternal nutrition, metabolism, muscle

## Abstract

Poor maternal nutrition during gestation negatively affects offspring growth and metabolism. To evaluate the impact of maternal nutrient restriction and realimentation on metabolism in the fetal liver, skeletal muscle, and circulation, on day 50 of gestation, ewes (*n* = 48) pregnant with singletons were fed 100% (CON) or 60% (RES) of requirements until day 90 of gestation, when a subset of ewes (*n* = 7/treatment) were euthanized, and fetal samples were collected. The remaining ewes were maintained on a current diet (CON-CON, *n* = 6; RES-RES, *n* = 7) or switched to an alternative diet (CON-RES, RES-CON; *n* = 7/treatment). On day 130 of gestation, the remaining ewes were euthanized, and fetal samples were collected. Fetal liver, longissimus dorsi (LD), and blood metabolites were analyzed using LC-MS/MS, and pathway enrichment analysis was conducted using MetaboAnalyst. Then, 600, 518, and 524 metabolites were identified in the liver, LD, and blood, respectively, including 345 metabolites that were present in all three. Nutrient restriction was associated with changes in amino acid, carbohydrate, lipid, and transulfuration/methionine metabolic pathways, some of which were alleviated by realimentation. Fetal age also affected metabolite abundance. The differential abundance of metabolites involved in amino acid, methionine, betaine, and bile acid metabolism could impact fetal epigenetic regulation, protein synthesis, lipid metabolism, and signaling associated with glucose and lipid metabolism.

## 1. Introduction

Inadequate nutrition during gestation impairs fetal growth and metabolism [1,2,3,4]. Impaired tissue growth during prenatal development can extend into early postnatal growth and through adulthood, thereby hindering the animal’s ability to develop adequate muscle. Maternal nutrient restriction during gestation causes metabolic dysregulation and alters key metabolic pathways in offspring [5,6,7,8]. Metabolism in skeletal muscle and liver tissues is susceptible to changes in nutrient supply, and restricted maternal nutrition can have detrimental impacts on development and metabolic activity in these tissues. Specifically, undernutrition has been reported to alter the abundance of metabolites involved in lipid and amino acid metabolism in the skeletal muscle of sheep offspring [9]. Similarly, maternal nutrient restriction increased lipid accumulation and altered factors related to glucose metabolism, fatty acid synthesis, and cholesterol metabolism [10,11,12,13]. These changes in the metabolic function of tissues prenatally can have adverse health and growth outcomes postnatally [14,15].

Following nutrient deprivation, restoring nutrients to adequate intake levels (or realimentation) is common practice and has been shown to induce compensatory growth in children, adults, and animals, which may alleviate the potentially adverse consequences of maternal nutrient restriction on offspring metabolism [16,17,18,19]. Realimentation following nutrient restriction may increase liver and muscle mass, as it has been shown to increase fat deposition in cattle, which could result in altered metabolism or the development of metabolic disease [20]. We demonstrated that maternal nutrient restriction reduced the muscle fiber cross-sectional area and liver weight during fetal development, and these effects were alleviated by realimentation [21].

Liver and muscle tissues contribute to the circulating metabolite pool, providing necessary nutrients such as glucose, fatty acids, and amino acids that can be utilized by other tissues in humans and livestock species [22,23]. Our previous study demonstrated altered lipid profiles in fetal muscle and liver in response to maternal nutrient restriction and realimentation [21]. Many studies have evaluated how maternal diet during gestation impacts offspring metabolism in various tissues. However, most studies evaluate metabolism in a single tissue or in circulation, while the evaluation of multiple tissues in conjunction with the circulation can provide a clearer understanding of alterations in systemic metabolism. The objective of this study was to evaluate the impacts of maternal nutrient restriction and realimentation on metabolism in fetal liver, skeletal muscle, and circulation using metabolomics analysis. We hypothesized that maternal nutrient restriction during gestation would impact metabolites in the fetal liver, longissimus dorsi and blood, and that realimentation would partially alleviate the effects of nutrient restriction. We used a sheep model as sheep have similar metabolic functions to humans, as well as nutrient transport to the fetus [24].

## 2. Materials and Methods

### 2.1. Experimental Design

All animal procedures were conducted at North Dakota State University (NDSU) as described previously [21,25,26,27]. Briefly, western whiteface ewe lambs (*n* = 90; < 1 year of age) were placed on pasture with three rams fitted with crayon marking harnesses. Singleton pregnancies were identified between days 30 and 40 of gestation using ultrasound. Forty-one ewes, pregnant with singletons, were housed in individual pens and fed a pelleted diet. On day 50 of gestation, a subset of ewes (*n* = 8) was euthanized, and fetal samples were collected. The remaining 41 ewes (mean initial body weight (BW) =  48.3  ±  0.6 kg) were assigned one of two dietary treatments—100% of nutrient requirements based on the National Research Council (NRC) recommendations (control; CON; *n* = 20) or 60% of NRC recommendations (restricted; RES; *n* = 21)—from day 50 to day 90 of gestation (mid-gestation). At day 90, 14 ewes were euthanized (CON, *n* = 7; RES, *n* = 7), and the remaining ewes were maintained on their current diet (CON-CON, *n* = 6; RES-RES, *n* = 7) or switched to the alternative diet (CON-RES, RES-CON; *n* = 7/treatment) from day 90 until day 130 of gestation, when they were euthanized for sample collection. Data for ewe body weight, fetal weight, placental weight, and organ weights have previously been reported [21,25,26,27].

### 2.2. Sample Collection

On day 90 (*n* = 14) and day 130 (*n* = 27), ewes were euthanized, and fetuses were extracted as described previously [25,26,27]. Fetal liver and longissimus dorsi samples were immediately snap-frozen in liquid nitrogen and stored at −80 °C. Fetal whole blood was collected via cardiac puncture and frozen at −20 °C. Samples were then shipped from NDSU to the University of Connecticut for analysis.

### 2.3. Metabolomic Analysis

#### 2.3.1. Sample Preparation

Fetal liver, longissimus dorsi, and whole blood samples (200 mg/sample tissue; 200 μL/blood sample; and the number of samples per treatment per tissue are presented in Table 1) were shipped on dry ice to Metabolon Inc. (Morrisville, NC, USA) for analysis. Samples were prepared using the automated MicroLab STAR system (Hamilton Company, Reno, NV). Several recovery standards were added prior to the first step in the extraction process for quality control purposes. To remove protein, dissociate small molecules bound to protein or trapped in the precipitated protein matrix, and recover chemically diverse metabolites, proteins were precipitated with methanol under vigorous shaking for 2 min (Glen Mills GenoGrinder 2000, SPEX Sample Prep, Metuchen, NJ, USA) followed by centrifugation. The resulting extract was divided into five fractions: two for analysis by two separate reverse-phase (RP)/ultra-high performance liquid chromatography (UPLC)–tandem mass spectrometry (MS/MS) methods with positive-ion-mode electrospray ionization (ESI); one for analysis by RP/UPLC-MS/MS with negative-ion-mode ESI; one for analysis by hydrophilic interaction chromatography (HILIC)/UPLC-MS/MS with negative-ion-mode ESI; and one sample for backup. Samples were placed briefly on a TurboVap^®^ (Zymark, Hopkinton, MA, USA) to remove the organic solvent. The sample extracts were stored overnight under nitrogen before preparation for analysis.

#### 2.3.2. Ultrahigh Performance Liquid Chromatography-Tandem Mass Spectroscopy (UPLC-MS/MS)

All methods utilized a Waters ACQUITY UPLC and a Thermo Scientific Q-Exactive high resolution/accurate mass spectrometer interfaced with a heated electrospray ionization source and Orbitrap mass analyzer operated at 35,000 (*m*/*z*)/Δ(*m*/*z*) mass resolution. The sample extract was dried then reconstituted in solvents compatible with each of the four methods. Each reconstitution solvent contained a series of standards at fixed concentrations to ensure injection and chromatographic consistency. One aliquot was analyzed using acidic positive-ion conditions, then chromatographically optimized for more hydrophilic compounds. In this method, the extract was gradient-eluted from a C18 column (Waters UPLC BEH C18-2.1 × 100 mm, 1.7 µm) using water and methanol, containing 0.05% perfluoropentanoic acid (PFPA) and 0.1% formic acid. Another aliquot was also analyzed using acidic positive-ion conditions; however, it was chromatographically optimized for more hydrophobic compounds. In this method, the extract was gradient-eluted from the same aforementioned C18 column using methanol, acetonitrile, water, 0.05% PFPA and 0.01% formic acid, which was operated at an overall greater organic content. Another aliquot was analyzed using basic negative-ion optimized conditions using a separate dedicated C18 column. The basic extracts were gradient-eluted from the column using methanol and water; however, this was with 6.5 mM ammonium bicarbonate at pH 8. The fourth aliquot was analyzed via negative ionization following elution from an HILIC column (Waters UPLC BEH Amide 2.1 × 150 mm, 1.7 µm) using a gradient consisting of water and acetonitrile with 10 mM ammonium formate at pH 10.8. Four separate quality controls were utilized and analyzed in concert with the experimental samples: a pooled matrix sample, generated by taking a small volume of each experimental sample (or alternatively, using a pool of well-characterized human plasma), served as a technical replicate throughout the data set; extracted water samples served as process blanks; and a cocktail of quality control standards, which were carefully chosen so as not to interfere with the measurement of endogenous compounds, were spiked in every analyzed sample, allowing for the monitoring of instrument performance and aiding in chromatographic alignment. Instrument variability was determined by calculating the median relative standard deviation for the standards that were added to each sample prior to injection into the mass spectrometers. Overall process variability was determined by calculating the median relative standard deviation for all endogenous metabolites (i.e., non-instrument standards) present in 100% of the pooled matrix samples. Experimental samples were randomized across the platform run, with quality control samples spaced evenly among the injections. The mass spectrometer (MS) analysis alternated between MS and data-dependent sequential mass spectrometry (MS^n^) scans using dynamic exclusion. The scan range varied slightly between methods but covered 70–1000 *m*/*z*.

#### 2.3.3. Data Extraction and Compound Identification

Raw data were extracted, peak-identified and quality control-processed using Metabolon’s hardware and software. Compounds were identified by comparison to library entries of purified standards or recurrent unknown entities. Metabolon maintains a library based on authenticated standards that contains the retention time/index mass-to-charge ratio (*m*/*z*) and chromatographic data (including MS/MS spectral data) on all molecules present in the library. Furthermore, biochemical identifications are based on three criteria: a retention index within a narrow retention time/index window of the proposed identification, an accurate mass match to the library +/− 10 ppm, and the MS/MS forward and reverse scores between the experimental data and authentic standards. The MS/MS scores are based on a comparison of the ions present in the experimental spectrum to the ions present in the library spectrum. While there may be similarities between these molecules based on one of these factors, all three data points can be utilized to distinguish and differentiate biochemicals. More than 3300 commercially available purified standard compounds have been acquired and registered into the LIMS for analysis on all platforms and for the determination of their analytical characteristics. Data were corrected by setting the median equal to one and normalizing each data point proportionately, and missing values were imputed to the minimum.

### 2.4. Statistical Analysis

All statistical analyses were conducted using R software (version 4.0.5; [28]). The metabolite fold-change was calculated as the ratio of metabolite means relative to CON (at day 90 of gestation) or CON-CON (at day 130 of gestation). The differential abundance of metabolites was determined by comparing the metabolite ratio values to one using *t*-tests, and statistical significance was determined at *p* < 0.05. Principal component analysis (PCA) was utilized to evaluate whether individuals clustered by treatment or day of gestation for each tissue, utilizing all identified metabolites. All differentially expressed metabolites were utilized for pathway analysis using MetaboAnalyst 5.0 (accessed on 12 May 2021 via: https://www.metaboanalyst.ca/), as described previously [29]. The ovine metabolite library is unavailable in MetaboAnalyst; therefore, the bovine metabolite library was utilized as there are many metabolic similarities between these species.

## 3. Results

### 3.1. Metabolites Identified

**Figure 1:**
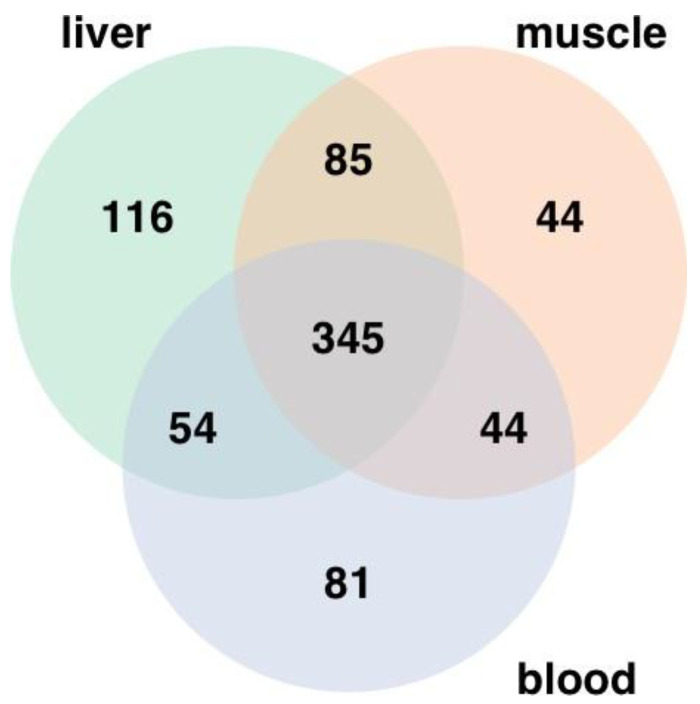
Distribution of metabolites identified in fetal liver (*n* = 41), longissimus dorsi muscle (*n* = 41), and blood (*n* = 38) tissues. A total of 600, 518, and 524 were identified in liver, longissimus muscle, and blood, respectively.

A total of 600, 518, and 524 metabolites were identified in the liver, longissimus dorsi, and blood, respectively, including 345 metabolites that were present in all three tissues (Figure 1).

**Figure 2:**
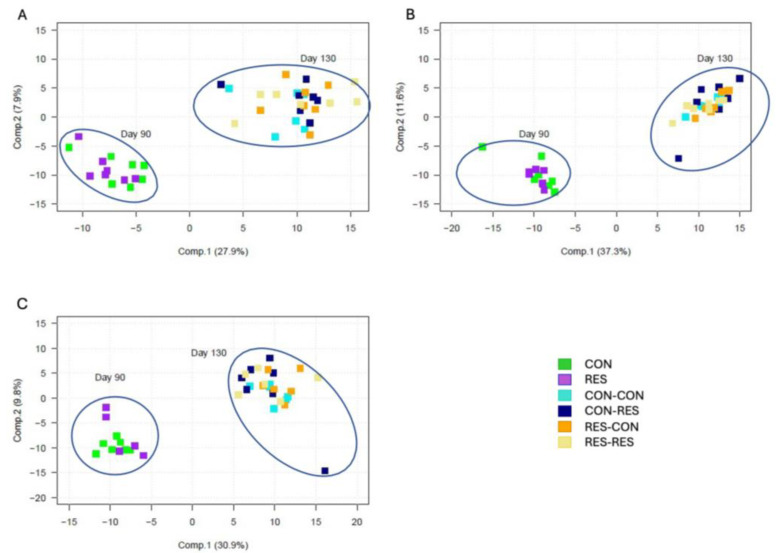
Principal component analysis of all metabolites detected in fetal (**A**) liver (*n* = 41), (**B**) longissimus dorsi (*n* = 41), and (**C**) blood (*n* = 38). Fetal samples were collected from fetuses of ewes at days 50, 90, and 130 of gestation and were analyzed by UPLC-MS/MS. CON = control day 25 to 90; RES = restricted day 50 to 90; CON-CON = control day 50 to 130; CON-RES = control day 50 to 90, restricted day 90 to 130; RES-CON = restricted day 50 to 90, control day 90 to 130; RES-RES = restricted day 50 to 130.

In the liver, PCA identified that principal components explained 32.36% and 8.17% of the variation, respectively (Figure 2A). In the longissimus dorsi, 44.24% and 12.20% of the variation was explained by principal components, respectively (Figure 2B). In the blood, principal components explained 36.85% and 11.04% of the variation, respectively (Figure 2C). The PCA demonstrated clustering of principal components by day of gestation in the liver, longissimus dorsi, and blood samples (Figure 2); however, differential metabolite abundances were also observed between treatments within the day of gestation.

### 3.2. Effects of Maternal Nutrient Restriction on Metabolic Pathways Specific to Tissue

**Figure 3:**
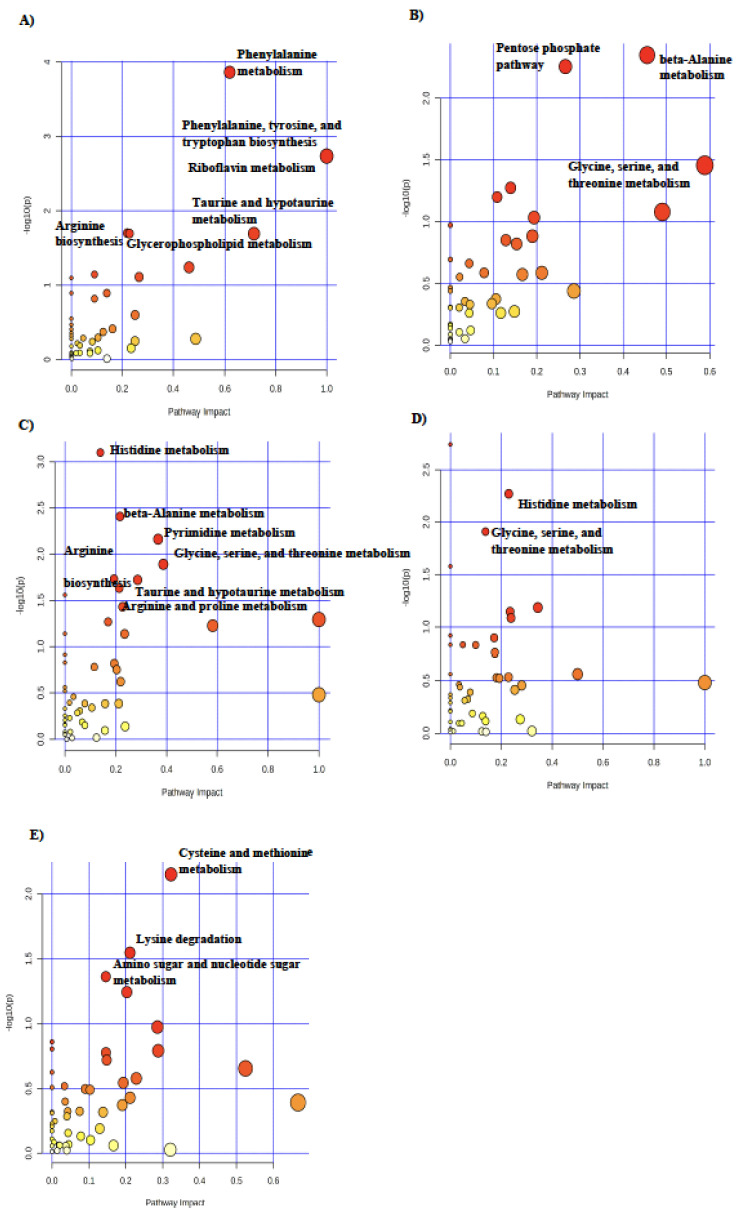
Pathway analysis of differential metabolites in fetal liver in (**A**) RES (*n* = 7) compared with CON (*n* = 7), (**B**) CON-RES (*n* = 7), (**C**) RES-RES (*n* = 7), and (**D**) RES-CON (*n* = 7) compared with CON-CON (*n* = 6), and (**E**) RES-CON compared with RES- RES. Circle colors are based on *p*-values (transition to red signifies decreasing *p*-value) and increasing circle size signifies increasing pathway impact. Pathway analysis was conducted in MetaboAnalyst 5.0. Significant pathways (*p* ≤ 0.05) with an impact greater than 0 are labeled.

**Figure 4:**
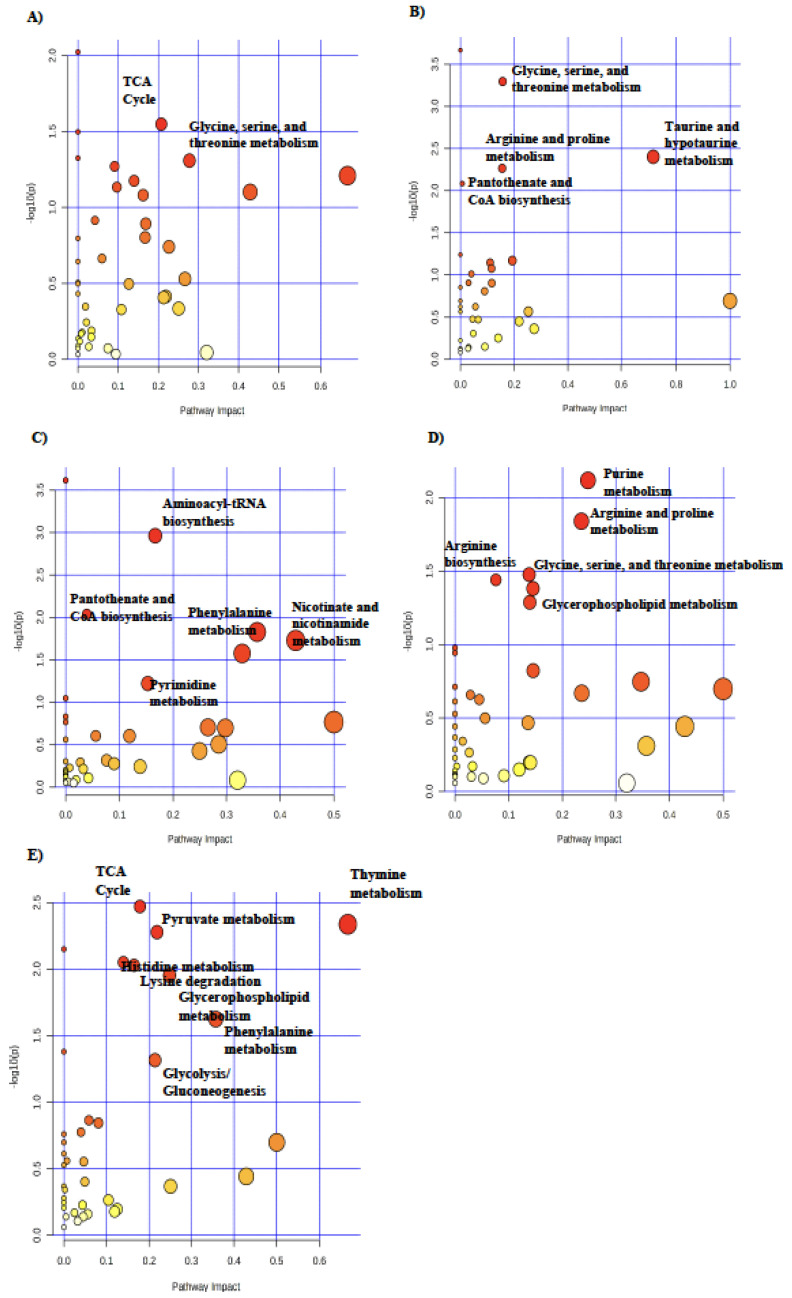
Pathway analysis of differential metabolites in fetal longissimus dorsi muscle in (**A**) RES (*n* = 7) compared with CON (*n* = 7) and (**B**) CON-RES (*n* = 7), (**C**) RES-RES (*n* = 7), and (**D**) RES-CON (*n* = 7) compared with CON-CON (*n* = 6) and (**E**) RES-CON compared with RES-RES. Circle colors are based on *p*-values (transition to red signifies decreasing *p*-value) and increasing circle size signifies increasing pathway impact. Pathway analysis was conducted in MetaboAnalyst 5.0. Significant pathways (*p* ≤ 0.05) with an impact greater than 0 are labeled.

**Figure 5:**
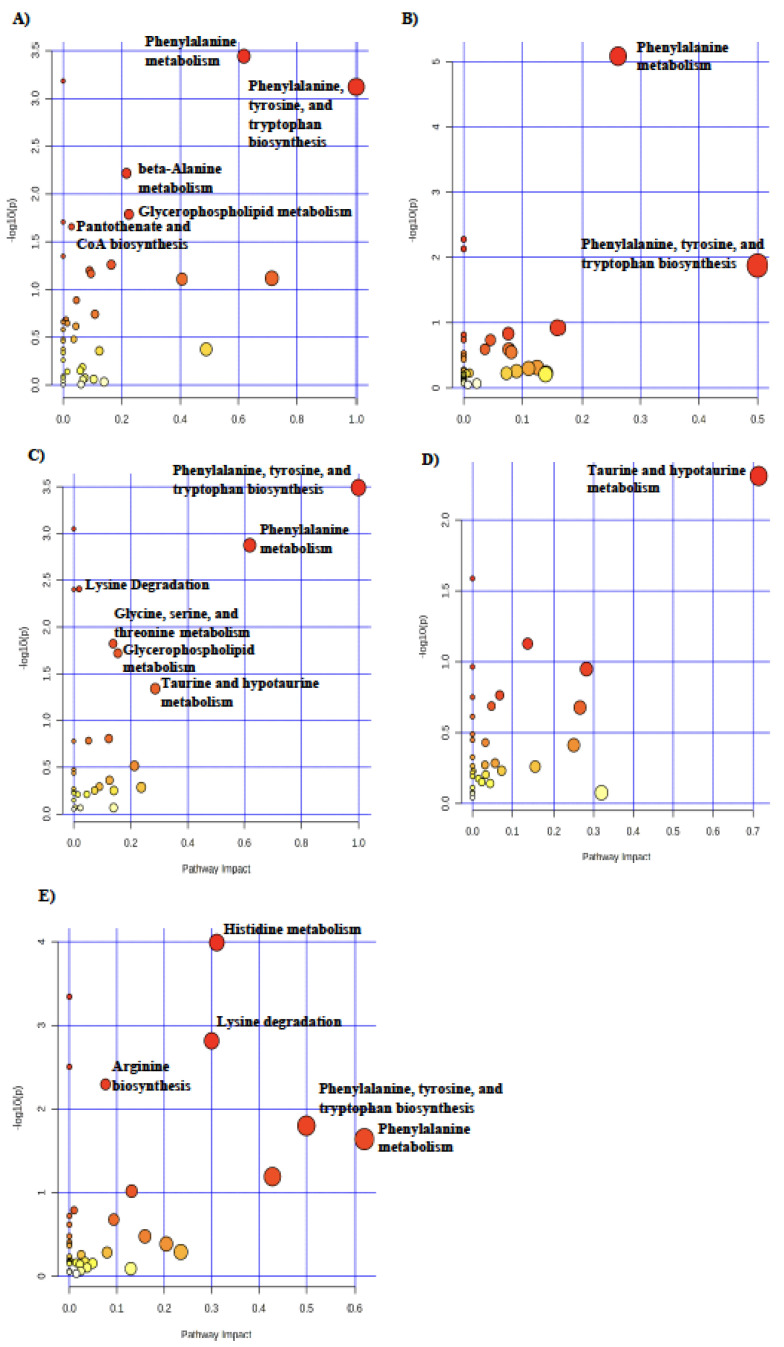
Pathway analysis of differential metabolites in fetal blood in (**A**) RES (*n* = 7) compared with CON (*n* = 7) and (**B**) CON-RES (*n* = 7), (**C**) RES-RES (*n* = 7), and (**D**) RES-CON (*n* = 7) compared with CON-CON (*n* = 6) and (**E**) RES-CON compared with RES-RES. Circle colors are based on *p*-values (transition to red signifies decreasing *p*-value) and increasing circle size signifies increasing pathway impact. Pathway analysis was conducted in MetaboAnalyst 5.0. Significant pathways (*p* ≤ 0.05) with an impact greater than 0 are labeled.

To determine the metabolic pathways impacted by maternal nutrition that were specific to each tissue, pathway analysis was conducted on the metabolites with differential abundance for each treatment within the liver, longissimus dorsi, and blood tissues (Figure 3, Figure 4 and Figure 5). In fetal liver, phenylalanine, tyrosine, and tryptophan biosynthesis was the most highly impacted pathway in RES compared with CON at day 90 of gestation (Figure 3A). Glycine, serine, and threonine metabolism was the most impacted pathway in CON-RES (Figure 3B) and RES-CON (Figure 3D). In the liver of RES-RES fetuses, histidine metabolism was the most impacted pathway (Figure 3C). In RES-CON, compared with RES-RES in the liver, cysteine and methionine metabolism was the most impacted pathway (Figure 3E).

In the fetal longissimus dorsi, glycine, serine, and threonine metabolism was the most impacted pathway in RES compared with CON at day 90 of gestation (Figure 4A). At day 130 of gestation, taurine and hypotaurine metabolism (Figure 4B), purine metabolism (Figure 4C), nicotinate and nicotinamide metabolism (Figure 4D), and thymine metabolism (Figure 4E) were the most highly impacted pathways in longissimus dorsi of CON-RES, RES-RES, RES-CON compared with CON-CON, and RES-CON compared with RES-RES, respectively.

In fetal blood, phenylalanine, tyrosine, and tryptophan biosynthesis was the most impacted pathway in RES compared with CON at day 90 of gestation (Figure 5A) as well as CON-RES (Figure 5B), and RES-CON (Figure 5D) was compared with CON-CON at day 130 of gestation. Taurine and hypotaurine metabolism was the most impacted pathway in RES-RES relative to CON-CON (Figure 5C). In RES-CON, compared with RES-RES, phenylalanine metabolism was the most impacted pathway in the blood (Figure 5E).

### 3.3. Effects of Maternal Nutrient Restriction at Day 90 of Gestation in Liver, Longissimus Dorsi, and Blood

**Figure 6:**
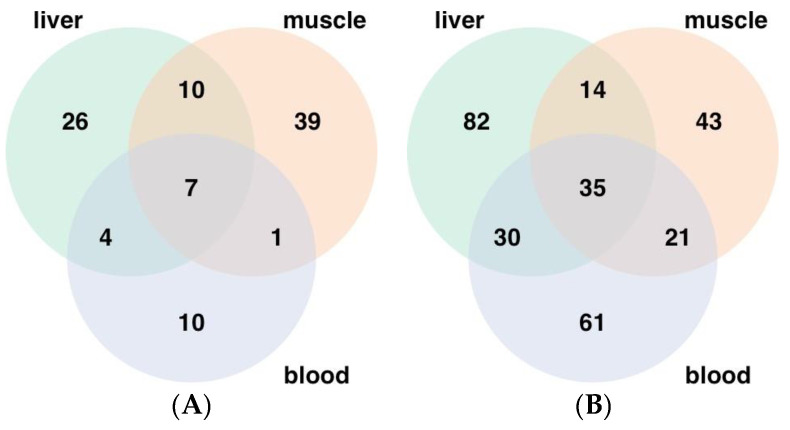
Distribution of differential metabolites (**A**) decreased and (**B**) increased in RES (restricted day 50 to 90 of gestation; *n* = 7) across fetal liver, longissimus dorsi muscle, and whole blood at day 90 of gestation compared with CON (control fed day 25 to 90 of gestation; *n* = 7).

To further evaluate the effects of the maternal diet on offspring metabolism and to gain clearer indications on metabolism throughout the whole body, metabolites with differential abundance across liver, longissimus dorsi, and blood tissues were identified. At day 90 of gestation, 208, 170, and 169 metabolites with differential abundances were identified in the liver, longissimus dorsi, and blood, respectively, in RES compared with CON (Figure 6). The distribution of decreased (Figure 6A) and increased (Figure 6B) metabolite abundances across each tissue is presented. Among the metabolites with differential abundances, 82 metabolites were decreased and 26 increased, specifically in the liver. Specific to the longissimus dorsi, 43 metabolites were decreased, and 39 metabolites were increased, and unique to the blood, 61 metabolites were decreased, and 10 metabolites were increased. Similar across all three tissues, 42 metabolites were found to have differential abundance in the liver, longissimus dorsi, and blood, with 35 metabolites decreased and 7 metabolites increased in RES compared with CON (Figure 6).

Table S1 depicts the common metabolites in the liver, longissimus dorsi, and blood that were differentially expressed in RES compared with CON at day 90 of gestation. In the RES offspring, the greatest number of metabolites with differential abundance across the three tissues were involved in amino acid/peptide metabolism. Specifically, the abundance of the branched-chain amino acid metabolites, valine, N-acetylvaline, gamma-glutamylvaline, isobutyrlglycine, 3-methylglutaconate, and 3-hydroxyisobutyrate were decreased in RES compared with CON (*p* ≤ 0.023; Table S1). Further, the abundance of histidine metabolites, 1-methylhistidine and 3-methylhistidine, were increased, and imidazole lactate was decreased in RES compared with CON at day 90 of gestation (*p* ≤ 0.045; Table S1). Several amino acid intermediates in lysine metabolism; methionine, cysteine, SAM and taurine metabolism; phenylalanine metabolism; and the urea cycle, arginine, and proline metabolism sub pathways were decreased in the liver, longissimus dorsi, and blood (*p* ≤ 0.047; Table S1), with the exception of phenylacetylglycine, which was increased in the liver, longissimus dorsi and blood in RES compared with CON (*p* ≤ 0.031; Table S1).

The differential carbohydrate metabolites identified across all three tissues were decreased in the liver, longissimus dorsi, and blood in RES compared with CON. Specifically, reductions were observed for fructose, arabonate, erythroante, mannitol, and N6-carboxymethyllysine (*p* ≤ 0.047; Table S1). Several lipid metabolites with differential abundance were observed in the liver, longissimus dorsi, and blood at day 90 of gestation (Table S1). Specifically, the bile acids, cholate and chenodeoxylcholate, decreased (*p* ≤ 0.002), and the taurine-conjugated bile acids, taurochenodeoxycholate, taurohyodeoxycholic acid, and tauroursodeoxycholate, were increased in RES relative to CON (*p* ≤ 0.046; Table S1). Choline phosphate was decreased in all three tissues in RES compared with the CON (*p* ≤ 0.01) liver, longissimus dorsi, and blood. Differential nucleotide, cofactors and vitamins, and xenobiotic metabolites that were identified in the liver, longissimus dorsi, and blood were decreased in RES compared with CON, except for methyl-4-hydroxybenzoate sulfate, which was increased in RES compared with CON in the liver, longissimus dorsi and blood (*p* ≤ 0.026; Table S1).

### 3.4. Effects of Maternal Diet in Liver, Longissimus Dorsi, and Blood at Day 130 of Gestation

Carnosine and anserine were increased, and ribulose 5-phosphate and xylulose 5-phosphate metabolites were decreased, in the liver in CON-RES, RES-CON, and RES-RES compared with CON-CON at day 130 of gestation (*p* ≤ 0.019; Table S2). In the *lo*ngissimus dorsi and blood, pregnanediol-3-glucuronide was increased in CON-RES, RES-CON, and RES-RES compared with CON-CON (*p* ≤ 0.039; Table S2). In the blood, taurochenodeoxycholic acid 3-sulfate and taurolithocholate 3-sulfate were increased, and 2-hydroxyphenylacetate, choline phosphate, and taurocholate were decreased in CON-RES, RES-CON, and RES-RES compared with CON-CON (*p* ≤ 0.030; Table S2).

### 3.5. Effects of Diet on Metabolites across All Tissues at Day 130 of Gestation

**Figure 7:**
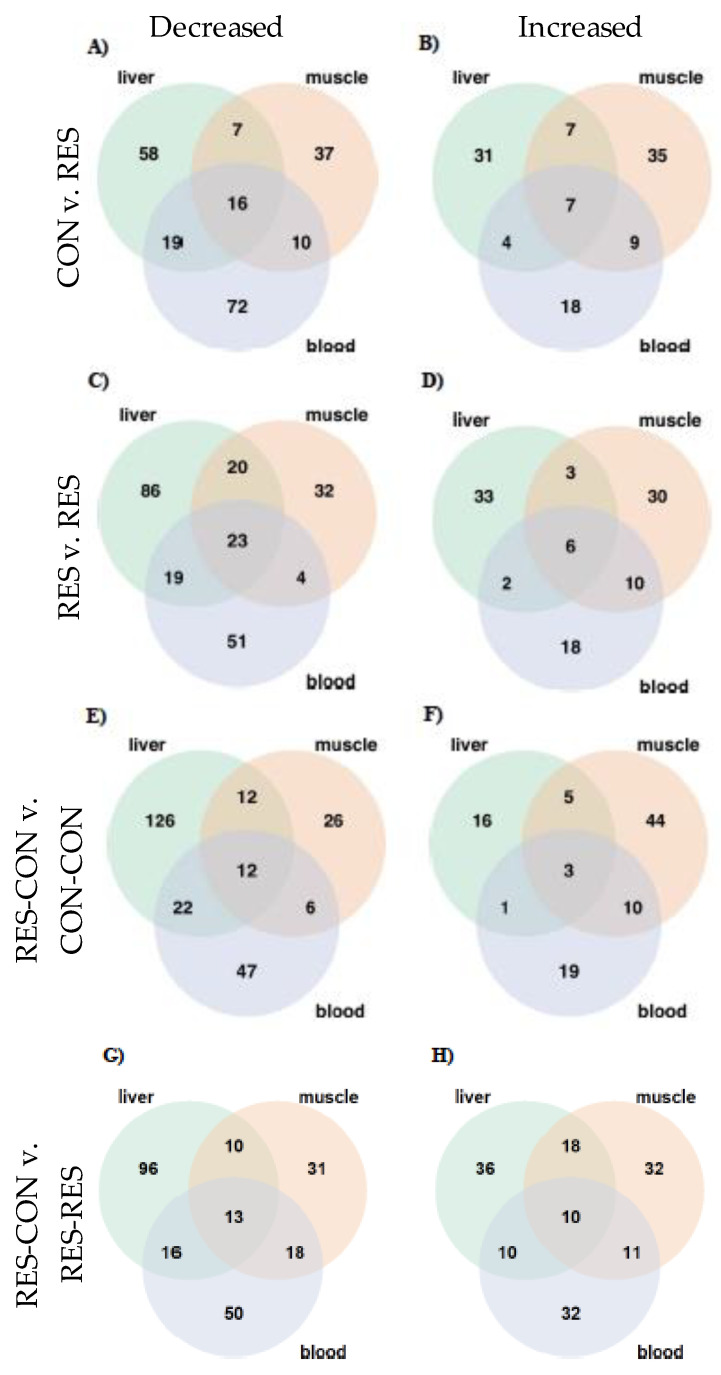
Distribution of differential metabolites (**A**) decreased and (**B**) increased in CON-RES, (**C**) decreased and (**D**) increased in RES-RES, (**E**) decreased and (**F**) increased in RES-CON compared with CON-CON, (**G**) decreased and (**H**) increased in RES-CON compared with RES- RES across fetal liver, muscle, and blood at day 130 of gestation.

The distribution of metabolites with differential abundance in CON-RES compared with CON-CON demonstrates that 16 metabolites were significantly decreased (Figure 7A) and 7 increased (Figure 7B) across liver, longissimus dorsi, and blood tissues. The majority of metabolites that exhibited differential abundance were lipids and amino acids in liver, longissimus dorsi, and blood tissues (Table S3). Specifically, the bile acids cholate, glycocholate, and glycodeoxycholate were decreased in the three tissues in CON-RES compared with CON-CON (*p* ≤ 0.01; Table S3). The carnitine metabolites carnitine, oleoylcarnitine (C18:1), palmitoleoylcarnintine (C16:1), and stearoylcarnitine (C18) were increased in CON-RES compared with CON-CON across the three tissues (*p* ≤ 0.046). The threonine metabolites threonine, gamma-glutamylthreonine, and sarcosine were decreased in CON-RES in each of the three tissues compared with CON-CON (*p* ≤ 0.019). With the exception of 2-hydroxybutyrate/2-hydroxyisobutyrate, which increased, the remaining arginine, proline, methionine, and glutathione metabolites were decreased in CON-RES compared with CON-CON in liver, longissimus dorsi, and blood tissues (*p* ≤ 0.026). In RES-RES at day 130 of gestation, 23 metabolites were decreased (Figure 7C) and 6 were increased (Figure 7D) compared with CON-CON. The glycine metabolites betaine and dimethylglycine were decreased in liver, longissimus dorsi, and blood tissues in RES-RES compared with CON-CON (*p* ≤ 0.028; Table S5). The amino acid metabolites 1-methylhistidine, 2-aminoadipate, 2- hydroxyadipate, 2-hydroxybutyrate/2-hydroxyisobutyrate, octadecanedioate (C18-DC), and urea were increased in RES-RES fetal liver, longissimus dorsi, and blood tissues compared with CON-CON (*p* ≤ 0.046; Table S5). Metabolites involved in phenylalanine, histidine, lysine, proline, and threonine were decreased in RES-RES compared with CON-CON in the three tissues (*p* ≤ 0.039). The bile acid metabolites cholate, glycocholate, and glycodeoxcholate were decreased in RES-RES compared with CON-CON in the liver, longissimus dorsi and blood (*p* ≤ 0.011). The lipid metabolites 2-aminoheptanoate, 2-hydroxyadipate, and choline phosphate were decreased in the liver, longissimus dorsi, and blood of RES-RES fetuses compared with CON-CON at day 130 of gestation (*p* ≤ 0.039). Metabolites involved in nucleotide metabolism and xenobiotics were decreased in the liver, longissimus dorsi, and blood in RES-RES compared with CON-CON (*p* ≤ 0.039: Table S5).

In the liver, longissimus dorsi, and blood of RES-CON relative to CON-CON, 12 metabolites were decreased (Figure 7E) and 3 metabolites were increased in common across the three tissues. (Figure 7F). Metabolites involved in amino acid metabolism had the greatest number of metabolites altered by mid-gestational nutrient restriction followed by realimentation (Table S4). Metabolites involved in arginine, glutamine, glycine, histidine, proline, threonine, and tryptophan metabolism were decreased in the liver, longissimus dorsi, and blood in RES-CON compared with CON-CON at day 130 of gestation (*p* ≤ 0.048; Table S4). Furthermore, the carbohydrate erythornate, the xenobiotic metabolite N-methylpipecolate, and the cofactor and vitamin metabolite trigonelline (N’-methylnicotinate) were increased, and taurohyodeoxycholic acid, a bile acid, was decreased, in the liver, longissimus dorsi, and blood in RES-CON compared with CON-CON (*p* ≤ 0.046: Table S4).

In the liver, longissimus dorsi, and blood of RES-CON relative to RES-RES, 13 metabolites were decreased (Figure 7G) and 10 metabolites were increased in common across the three tissues (Figure 7H). Metabolites involved in amino acid metabolism and lipid metabolism had the greatest number of metabolites with differential abundance in RES-CON compared with RES-RES. Specifically, histidine metabolites, 1-methylhistidine, 3-methylhistidine, and imidazole propionate were decreased in RES-CON compared with RES-RES in the liver, longissimus dorsi, and blood. (*p* ≤ 0.018; Table S6). Metabolites involved in the metabolism of lysine, glutathione, glutamate, and glycine were decreased in RES-CON compared with RES-RES in all three tissues (*p* ≤ 0.042; Table S6). The lipid metabolites taurohyodeoxycholic acid and trimethylamine N-oxide were decreased and increased, respectively, in the liver, longissimus dorsi, and blood (*p* ≤ 0.041; Table S6). Furthermore, carbohydrate metabolites, erythronate, fructose, and N6-carboxymethylysine were increased in all three tissues (*p* ≤ 0.042; Table S6), and xenobiotic metabolites involved in benzoate metabolism were increased in all three tissues of RES-CON relative to RES-RES (*p* ≤ 0.026; Table S6).

## 4. Discussion

Maternal nutrient restriction during gestation can alter metabolism in the offspring, which can increase the risk of developing metabolic disease and obesity later in life [14,15,30]. Realimentation can increase the growth rate of nutrient-restricted animals, which may be due to changes in metabolism; however, the effects of maternal realimentation on offspring metabolism are not well understood [16,17,18]. The objective of this study was to evaluate the effects of maternal nutrient restriction followed by realimentation on fetal metabolism in liver, longissimus dorsi, and blood tissues. We utilized sheep as they are a well-established model of maternal nutrient restriction and exhibit similar metabolic and growth outcomes to humans in response to maternal nutrient restriction [24,31].

The liver and skeletal muscles are key metabolic tissues and have integral roles in maintaining glucose homeostasis, lipid metabolism, protein turnover, and amino acid regulation and contribute significantly to the circulating metabolite pool, which can be utilized by other tissues in the body [22,23,32,33]. The evaluation of metabolism in multiple tissues provides the opportunity to elucidate key metabolic pathways altered by maternal diet that may systemically impact development. To determine the effects of nutrient restriction and realimentation on metabolism in the whole system, metabolomic analysis was conducted in liver, longissimus dorsi, and blood tissues, and metabolites with differential abundance that were common to all three tissues were identified. Compared with controls, the number of metabolites with differential abundance in liver, longissimus dorsi, and blood tissues was smallest in the realimented group in our study. Compared with continuously restricted animals, there were more metabolites identified in the realimented group that exhibited differential abundance compared with controls. This suggests that realimentation can alleviate changes in overall metabolite concentrations following nutrient restriction and result in a metabolic profile similar to controls. Pathway enrichment analysis demonstrated that alterations in metabolism in the realimented group relative to the continuous restricted group can impact the amino acid metabolic pathways. The fetal liver of the realimented group had the most metabolites with differential abundance identified among all the tissues, indicating that realimentation may be beneficial in alleviating changes in metabolism in multiple tissues but has a unique effect in arginine and proline metabolism and pyrimidine metabolism in the liver. Interestingly, as previously reported [21,25,26,27], differences in fetal body weight and organ weights were not detected in response to maternal diet; however, the changes in metabolic profiles suggest that these contribute to the persistent metabolic dysregulation observed in the offspring of poorly fed mothers.

Maternal undernutrition has been shown to increase fat accumulation in the liver [10] and longissimus dorsi [34] and alter the abundance of lipid metabolites in the longissimus dorsi [21,35]. In our study, we report increased carnitine metabolite abundances in the liver, longissimus dorsi, and blood in response to late-gestational nutrient restriction. Carnitine is involved in fatty acid oxidation by facilitating the transport of long-chain fatty acids into the mitochondria for subsequent β-oxidation [35,36,37]. In mammals, the fetus is unable to synthesize endogenous carnitine [38]. Therefore, alterations in carnitine and acyl-carnitine concentrations are likely due to increased carnitine supply from the ewe to the developing fetus across the placenta. However, further studies are necessary to confirm this hypothesis. 

The developing fetus relies primarily on carbohydrates and amino acids for the production of energy and protein synthesis during gestation in human and animal species [39]. In this study, urea concentrations were increased in fetal liver, muscle, and blood due to long-term nutrient restriction. During periods of starvation or reduced nutrition in humans and livestock, proteins are catabolized to promote energy production, resulting in the production of toxic ammonia [40]. The toxic ammonia is converted to urea via the urea cycle for excretion in the urine [40]. Increases in urea in tissues indicates the increased catabolism of amino acids, transforming the carbon skeletons of amino acids into other intermediates, such as glucose [41]. In this study, most of the top-enriched pathways due to nutrient restriction involved the metabolism of amino acids—specifically, cysteine and methionine, lysine, phenylalanine, and branched-chain amino acid. These results suggest an increased utilization of amino acids as metabolic precursors for energy production to promote growth. Realimentation also influenced the abundance of metabolites involved in amino acid metabolism in the current study. Specifically, in the liver, muscle, and blood, decreases in the essential amino acids methionine and threonine were observed. These data are consistent with previously published data where a reduction in methionine and threonine concentrations was observed in the fetal plasma of the offspring of nutrient-restricted ewes that were realimented on day 78 of gestation [42]. In ruminants, the majority of essential amino acids are produced by the metabolism of non-protein nitrogen by rumen microbes, which can support reduced growth even if they are deficient in the diet [43]. In addition to the importance of methionine in protein synthesis, methionine also acts as a methyl donor for DNA methylation [44,45]. Therefore, the decrease in methionine concentrations may impact whole-body epigenetic regulation. This suggests that realimentation influences local and systemic amino acid metabolism, which may impact amino acid regulation and protein synthesis postnatally and alter epigenetic regulation through DNA methylation.

In our experiment, in fetal liver, muscle, and blood, there was a decrease in betaine due to mid–late gestational nutrient restriction. Betaine is the trimethyl derivative of glycine and can be obtained from the diet or generated by the oxidation of choline [46]. Betaine is involved in several metabolic processes including amino acid sparing, osmotic stress protection, hepatic lipid mobilization, and fat distribution and has been shown to promote growth when used as a dietary supplement in adolescent pigs, sheep, and poultry [46,47,48,49]; however, knowledge on the effects of nutrient restriction on betaine concentrations in fetal tissues is limited. Due to the involvement of betaine in one-carbon metabolism and epigenetic regulation, it has an important role in fetal development. Specifically, betaine acts as a methyl-donor involved in the conversion of homocysteine to methionine and, therefore, is a vital source of methyl groups for maintaining methylation reactions during embryonic and fetal development [49]. Betaine is also widely used as a feed additive to promote growth in livestock and has been studied as a supplement during gestation that can impact fetal growth [50,51,52,53,54]. Specifically, in pigs, the offspring of mothers supplemented with dietary betaine had increased birth weights, and reduced insulin-like growth factor-1 expression was found in rats [53,54]. Betaine supplementation during gestation has also been shown to impact metabolism by influencing the expression of genes involved in lipogenesis, cholesterol metabolism, and gluconeogenesis through epigenetic modifications in the livers of offspring in pigs and rats [50,51,52,54]. In swine, betaine supplementation improves growth, meat quality, and intramuscular fat [55]. These results indicate that betaine is integral for several processes promoting growth and metabolism and that the reduction in betaine, due to maternal nutrient restriction, may influence the epigenetic regulation of metabolism in the offspring. In addition, the product of betaine metabolism, dimethylglycine, was also reduced in the liver, muscle, and blood due to mid-to-late gestational nutrient restriction at day 130 of gestation. This could be a direct result of reduced transmethylation of betaine to homocysteine. Dimethylglycine was also reduced by nutrient restriction during mid-gestation followed by realimentation in fetal liver, muscle, and blood tissues at day 130 of gestation, but no difference in betaine was observed in this study. The decrease in dimethylglycine may be correlated with the reduction in methionine; however, further investigation is needed to confirm this hypothesis.

Homoarginine was reduced in all three tissues in response to restricted nutrition during late gestation (CON-RES) and nutrient restriction during mid-gestation followed by realimentation (RES-CON) in the present study. Recent epidemiological studies in humans have linked decreased homoarginine concentrations with an increased risk of cardiovascular disease [56]. Therefore, it is theorized that homoarginine may have protective cardiovascular effects. Epidemiological studies in humans have also linked poor maternal nutrition and low birth weight with an increased risk of developing cardiovascular disease [14]. Therefore, the reduced homoarginine observed in our study could be an indicator of cardiovascular disease later in life, if this phenotype persists.

The pentose phosphate pathway is involved in producing NADPH, a co-factor for antioxidant enzymes, nitric oxide synthase, and various lipid synthesis enzymes, as well as ribose 5-phosphate, a precursor for nucleotide biosynthesis [57,58,59]. Knowledge on the effects of maternal nutrition on the pentose phosphate pathway is limited; however, this study observed that nutrient restriction reduced the pentose phosphate metabolites ribulose 5-phosphate and xylulose 5-phosphate in the liver at day 130 of gestation. Pathway analysis showed that the pentose phosphate pathway was impacted in the liver in CON-RES, suggesting that nutrient restriction during late gestation impacts the pentose phosphate pathway, which may impact carbohydrate metabolism postnatally.

## 5. Conclusions

Metabolism is a complex system of chemical reactions required for the survival of the cell or tissue [60]. Tissues can have unique metabolic functions and constantly take up from and release nutrients and other factors to the circulating pool of metabolites [61]. We and others have evaluated the impacts of maternal nutrient restriction on offspring metabolism in specific tissues such as the muscle and liver and in blood circulation [6,9,12,13,61,62]. In the present study, using a metabolomic analysis, in the liver, muscle, and blood, we were able to identify alterations in metabolism in multiple tissues, which is important for understanding the impact of maternal nutrient restriction and realimentation on offspring growth, development, and metabolism throughout the whole body. The liver, skeletal muscle, and blood have integral roles in local and systemic metabolism, and differential abundances of metabolites involved in the metabolism of methionine, betaine, and bile acids were observed. These differential metabolites could impact epigenetic regulation, protein synthesis, lipid metabolism, and the signaling pathways of glucose and lipid metabolic factors in the fetus. These alterations in metabolism may impair offspring growth postnatally, which may lead to metabolic dysregulation throughout life. Furthermore, this research could be used to identify biomarkers to develop potential management strategies for alleviating changes in fetal development in response to maternal nutrient restriction. Further research is necessary to determine if these alterations in metabolites persist postnatally and into adulthood. We recognize the limitations of our study. For example, due to limited information on ovine metabolites, a bovine metabolite library was utilized. This could have prevented us from identifying all metabolites unique to sheep or in response to dietary changes. However, the bovine and sheep genomes are very similar and, therefore, we expect that since they have similar digestion and metabolic processes, the findings in the current study can be applied to sheep. Future studies would benefit from more in-depth analysis of specific metabolites to confirm the response to maternal diet in each tissue as well as the use of metabolites as feed additives to improve growth and metabolism in response to periods of poor maternal nutrition or other stressors during gestation.

## Figures and Tables

**Table 1:** Distribution of samples per treatment utilized in metabolomic analysis.

	*n*			
Diet ^1^	Liver	LD	Blood	Description
CON	7	7	7	
RES	7	7	5 ^†^	Restricted diet maintained from day 50 to day 90
CON-CON	6	6	6	Control diet maintained through day 130
CON-RES	7	7	7	Control diet switched to restricted diet days 90 through 130
RES-CON	7	7	7	Restricted diet switched to control diet days 90 through 130
RES-RES	7	7	6 ^†^	Restricted diet maintained from day 50 to day 130

^1^ Abbreviations: CON = control day 25 to 90; RES = restricted day 50 to 90; CON-CON = control day 50 to 130; CON-RES = control day 50 to 90, restricted day 90 to 130; RES-CON = restricted day 50 to 90, control day 90 to 130; RES-RES = restricted day 50 to 130. ^†^ We were unable to be collect sufficient volume of fetal blood for metabolomic analysis for two animals in RES and one in RES-RES.

## Data Availability

The data presented in this study are available on request from the corresponding author due to continued data analysis for additional research objectives.

## Supplementary Materials

The following supporting information can be downloaded at: https://www.mdpi.com/article/10.3390/metabo14090465/s1, Table S1: Differential metabolites common across liver, muscle, and blood in RES at day 90; Table S2: Differential metabolites across all treatments in liver, muscle, and blood at day 130 of gestation; Table S3: Differential metabolites common across liver, muscle, and blood in CON-RES at day 130 of gestation; Table S4: Differential metabolites common across liver, muscle, and blood in RES-CON at day 130 of gestation; Table S5: Differential metabolites common across liver, muscle, and blood in RES-RES at day 130 of gestation; Table S6: Differential Metabolites common across liver, muscle, and blood in RES-CON relative to RES-RES at day 130 of gestation.
