# Supplementary material for: The Effects of Maternal Nutrient Restriction during Mid to Late Gestation with Realimentation on Fetal Metabolic Profiles in the Liver, Skeletal Muscle, and Blood in Sheep
Source: Metabolites. 2024 Aug 23;14(9):465. doi: 10.3390/metabo14090465 (PMC11434268; doi:10.3390/metabo14090465)
Supplement: Supplementary file 1 [file metabolites-14-00465-s001.zip › metabolites-3140447-supplementary.pdf]

Table S1: Differential metabolites common across liver, muscle, and blood in RES at day 90.

			Fold Change			*P*-value		
Super Pathway	Sub Pathway	Metabolite	Liver	Muscle	Blood	Liver	Muscle	Blood
Amino Acid/Peptide								
	Histidine Metabolism	1-methylhistidine	1.33	1.55	1.37	<0.001	<0.001	0.019
	Leucine, Isoleucine and Valine Metabolism	3-hydroxyisobutyrate	-1.28	-1.39	-1.64	0.012	<0.001	<0.001
	Leucine, Isoleucine and Valine Metabolism	3-methylglutaconate	-1.35	-1.27	-1.45	0.007	0.021	0.023
	Histidine Metabolism	3-methylhistidine	1.63	1.42	3.57	<0.001	<0.001	0.045
	Gamma-glutamyl Amino Acid	gamma-glutamylphenylalanine	-1.22	-1.27	-1.18	<0.001	<0.001	0.030
	Gamma-glutamyl Amino Acid	gamma-glutamylvaline	-1.18	-1.35	-1.64	0.007	<0.001	<0.001
	Histidine Metabolism	imidazole lactate	-1.16	-1.39	-1.28	0.015	<0.001	0.047
	Leucine, Isoleucine and Valine Metabolism	isobutyrylglycine	-1.37	-1.72	-1.41	0.011	0.004	0.014
	Leucine, Isoleucine and Valine Metabolism	N-acetylvaline	-1.72	-1.16	-1.28	0.003	0.009	0.023
	Urea cycle; Arginine and Proline Metabolism	N-methylproline	-3.57	-3.70	-4.17	<0.001	<0.001	<0.001
	Lysine Metabolism	N6,N6-dimethyllysine	-1.19	-1.32	-1.30	0.026	0.002	0.017
	Lysine Metabolism	N6-methyllysine	-1.18	-1.27	-1.37	0.006	<0.001	0.003
	Acetylated Peptides	phenylacetylglycine	1.73	1.77	1.58	0.002	<0.001	0.031
	Phenylalanine Metabolism	phenyllactate (PLA)	-1.22	-1.39	-1.64	0.031	0.004	0.011

	Methionine, Cysteine, SAM and Taurine Metabolism	S-methylcysteine sulfoxide	-1.18	-1.22	-1.27	0.040	0.002	0.031
	Urea cycle; Arginine and Proline Metabolism	trans-4-hydroxyproline	-1.11	-1.06	-1.28	0.021	0.013	0.047
	Leucine, Isoleucine and Valine Metabolism	valine	-1.30	-1.23	-1.67	0.001	<0.001	<0.001
Carbohydrate								
	Pentose Metabolism	arabonate/xylonate	-1.20	-1.16	-1.18	0.006	0.047	0.004
	Aminosugar Metabolism	erythronate	-1.19	-1.18	-1.30	0.003	<0.001	0.010
	Fructose, Mannose and Galactose Metabolism	fructose	-1.20	-1.25	-1.32	0.007	<0.001	0.005
	Fructose, Mannose and Galactose Metabolism	mannitol/sorbitol	-1.27	-1.18	-1.22	0.003	0.014	0.030
	Advanced Glycation End-product	N6-carboxymethyllysine	-1.30	-1.37	-1.49	0.006	<0.001	0.007
Lipid								
	Progestin Steroids	5alpha-pregnan-3beta,20alpha-diol monosulfate	-1.64	-1.49	-2.78	0.009	0.045	<0.001
	Primary Bile Acid Metabolism	chenodeoxycholate	-1.43	-2.27	-1.89	<0.001	<0.001	0.002
	Primary Bile Acid Metabolism	cholate	-33.33	-50.00	-33.33	<0.001	<0.001	<0.001
	Phospholipid Metabolism	choline phosphate	-1.09	-1.08	-1.49	0.008	0.005	0.010

	Primary Bile Acid Metabolism	taurochenodeoxycholate	2.19	1.89	1.63	<0.001	0.005	0.007
	Secondary Bile Acid Metabolism	taurohyodeoxycholic acid	2.33	1.72	1.38	0.002	0.004	0.039
	Secondary Bile Acid Metabolism	tauroursodeoxycholate	3.58	3.71	2.79	0.006	0.003	0.046
	Phospholipid Metabolism	trimethylamine N-oxide	-2.50	-2.56	-3.85	<0.001	<0.001	<0.001
Nucleotide								
	Pyrimidine Metabolism, Uracil containing	5,6-dihydrouridine	-1.20	-1.28	-1.43	0.009	0.001	0.013
	Purine Metabolism, (Hypo)Xanthine/Inosine containing	allantoin	-1.52	-1.54	-1.85	0.003	<0.001	0.004
	Pyrimidine Metabolism, Uracil containing	pseudouridine	-1.22	-1.20	-1.30	0.001	<0.001	0.026
Cofactors and Vitamins								
	Nicotinate and Nicotinamide Metabolism	trigonelline (N'-methylnicotinate)	-5.00	-3.33	-3.85	<0.001	<0.001	<0.001
Xenobiotics								
	Benzoate Metabolism	2-hydroxyhippurate (salicylurate)	-1.45	-1.64	-1.69	<0.001	<0.001	0.004
	Benzoate Metabolism	4-ethylphenylsulfate	-1.43	-1.61	-1.75	<0.001	<0.001	0.007
	Food Component/Plant	homostachydrine	-1.79	-1.64	-1.85	<0.001	0.001	0.002
	Food Component/Plant	mannonate*	-1.27	-1.22	-1.41	<0.001	<0.001	<0.001

Benzoate Metabolism	methyl-4-hydroxybenzoate sulfate	1.62	1.58	1.36	0.024	0.011	0.019
Bacterial/Fungal	N-methylpipecolate	-1.67	-1.75	-1.64	<0.001	<0.001	0.004
Food Component/Plant	pyrraline	-1.72	-2.56	-3.03	<0.001	<0.001	<0.001
Food Component/Plant	stachydrine	-2.56	-2.27	-2.44	<0.001	<0.001	0.002

Note: Fold change is relative to CON at day 90 of gestation in each tissue. Calculated relative fold change values were compared to 1 using t-tests. Significance denoted at $P \leq 0.05$.
Abbreviations: RES = restricted day 50 to 90; CON = control day 50 to 90

Table S2: Differential metabolites across all treatments in liver, muscle, and blood at day 130 of gestation.

		Fold Change			*P*-value		
Tissue	Metabolites	CON-RES	RES-CON	RES-RES	CON-RES	RES-CON	RES-RES
Liver							
	anserine	1.51	1.46	1.63	0.013	0.001	0.007
	carnosine	1.30	1.56	1.43	0.019	<0.001	0.006
	ribulose 5-phosphate	-2.38	-1.82	-1.64	0.006	<0.001	0.002
	xylulose 5-phosphate	-1.54	-1.54	-1.47	<0.001	<0.001	<0.001
Muscle							
	pregnanediol-3-glucuronide	1.70	1.71	1.64	0.039	0.010	0.011
Blood							
	2-hydroxyphenylacetate	-1.41	-1.56	-1.49	0.002	<0.001	0.001
	choline phosphate	-1.28	-1.25	-1.35	0.003	0.004	0.002
	pregnanediol-3-glucuronide	1.40	1.40	1.32	0.005	0.003	0.020
	taurochenodeoxycholic acid 3-sulfate	3.49	3.35	3.38	0.006	0.006	0.012
	taurocholate	-2.08	-2.44	-2.78	<0.001	<0.001	<0.001
	taurolithocholate 3-sulfate	3.87	2.49	2.22	0.024	0.030	0.016

Note: Fold-change is relative to CON-CON at day 130 of gestation. Significance determined at $P \leq 0.05$.
Abbreviations: CON-CON = control day 50 to 130; CON-RES = control day 50 to 90, restricted day 90 to 130; RES-CON = restricted day 50 to 90, control day 90 to 130; RES-RES = restricted day 50 to 130 gestation.

Table S3: Differential metabolites common across liver, muscle, and blood in CON-RES at day 130 of gestation.

Super Pathway	Sub Pathway	Metabolite	Fold Change			*P*-value		
			Liver	Muscle	Blood	Liver	Muscle	Blood
Amino Acid/Peptide								
	Glutathione Metabolism	2-hydroxybutyrate/2-hydroxyisobutyrate	1.24	1.76	1.36	0.040	0.005	0.006
	Gamma-glutamyl Amino Acid	gamma-glutamylthreonine	-1.47	-2.13	-2.04	0.002	<0.001	<0.001
	Urea cycle; Arginine and Proline Metabolism	homoarginine	-1.33	-1.28	-1.41	0.001	0.019	<0.001
	Methionine, Cysteine, SAM and Taurine Metabolism	methionine sulfone	-1.25	-1.23	-1.20	0.004	0.003	0.026
	Urea cycle; Arginine and Proline Metabolism	N-delta-acetylornithine	-1.39	-1.64	-1.49	0.010	<0.001	0.001
	Urea cycle; Arginine and Proline Metabolism	N-methylproline	-1.61	-1.67	-1.67	0.003	0.002	0.002
	Glycine, Serine and Threonine Metabolism	sarcosine	-1.35	-1.52	-1.33	0.013	0.011	0.001
	Glycine, Serine and Threonine Metabolism	threonine	-1.27	-1.59	-1.61	0.002	<0.001	<0.001
Carbohydrate								
	Advanced Glycation End-product	N6-carboxymethyllysine	-1.35	-1.52	-1.43	<0.001	<0.001	0.002
Lipid								
	Fatty Acid, Amino	2-aminoheptanoate	-1.82	-1.89	-2.04	<0.001	<0.001	<0.001
	Fatty Acid, Dicarboxylate	2-hydroxyadipate	1.55	2.47	1.56	0.028	0.012	0.030
	Carnitine Metabolism	carnitine	1.31	1.20	1.51	0.002	<0.001	0.020
	Primary Bile Acid Metabolism	cholate	-4.76	-7.69	-11.11	<0.001	<0.001	<0.001
	Primary Bile Acid Metabolism	glycocholate	-1.49	-2.94	-1.67	0.010	<0.001	0.007

	Secondary Bile Acid Metabolism	glycodeoxycholate	-5.00	-2.63	-7.14	<0.001	0.002	<0.001
	Fatty Acid, Dicarboxylate	octadecenedioate (C18:1-DC)	1.48	2.14	1.84	0.008	<0.001	0.007
	Fatty Acid Metabolism (Acyl Carnitine, Monounsaturated)	oleoylcarnitine (C18:1)	1.58	1.67	1.67	0.041	0.020	0.012
	Fatty Acid Metabolism (Acyl Carnitine, Monounsaturated)	palmitoleoylcarnitine (C16:1)	1.64	1.69	1.60	0.046	0.016	0.028
	Fatty Acid Metabolism (Acyl Carnitine, Long Chain Saturated)	stearoylcarnitine (C18)	1.93	1.38	1.74	0.039	0.039	0.016
Nucleotide								
	Pyrimidine Metabolism, Uracil containing	5,6-dihydrouridine	-1.23	-1.25	-1.23	0.018	0.008	0.027
Xenobiotics								
	Benzoate Metabolism	2-hydroxyhippurate (salicylurate)	-2.17	-1.54	-2.13	0.001	0.006	<0.001
	Benzoate Metabolism	4-ethylphenylsulfate	-1.79	-1.32	-1.75	<0.001	0.050	<0.001
	Food Component/Plant	pyrraline	-1.37	-2.04	-1.79	0.007	<0.001	<0.001

Note: Fold change is relative to CON-CON at day 130 gestation in each tissue. Calculated relative fold change values were compared to 1 using t-tests. Significance denoted at $P \leq 0.05$.
Abbreviations: CON-RES = control day 50 to 90, restricted day 90 to 130 gestation; CON-CON = control day 50 to 130 gestation.

Table S4: Differential metabolites common across liver, muscle, and blood in RES-CON at day 130 of gestation.

Super Pathway	Sub Pathway	Metabolite	Fold Change			*P*-value		
			Liver	Muscle	Blood	Liver	Muscle	Blood
Amino Acid/Peptide								
	Glycine, Serine and Threonine Metabolism	dimethylglycine	-1.35	-1.32	-1.41	0.024	0.028	0.008
	Gamma-glutamyl Amino Acid	gamma-glutamylthreonine	-1.30	-1.45	-1.33	0.015	0.008	0.018
	Urea cycle; Arginine and Proline Metabolism	homoarginine	-1.35	-1.19	-1.27	0.008	0.019	0.021
	Histidine Metabolism	imidazole lactate	-1.52	-1.35	-1.61	<0.001	<0.001	<0.001
	Histidine Metabolism	imidazole propionate	-1.89	-1.54	-1.92	0.001	0.026	0.001
	Tryptophan Metabolism	indolelactate	-1.67	-1.15	-1.30	0.004	0.011	<0.001
	Methionine, Cysteine, SAM and Taurine Metabolism	methionine	-1.10	-1.19	-1.12	0.048	0.002	0.002
	Urea cycle; Arginine and Proline Metabolism	N-methylproline	-1.72	-1.61	-1.72	0.002	<0.001	<0.001
	Phenylalanine Metabolism	phenyllactate (PLA)	-1.54	-1.52	-1.41	<0.001	<0.001	<0.001
	Glutamate Metabolism	pyroglutamine	-1.14	-1.10	-1.12	0.017	0.011	0.041
	Glycine, Serine and Threonine Metabolism	threonine	-1.22	-1.25	-1.30	0.015	0.037	0.009
Carbohydrate								
	Aminosugar Metabolism	erythronate	1.27	1.37	1.22	0.046	0.008	0.038
Lipid								
	Secondary Bile Acid Metabolism	taurohyodeoxycholic acid	-1.69	-2.70	-3.13	<0.001	<0.001	<0.001

Cofactors and Vitamins								
	Nicotinate and Nicotinamide Metabolism	trigonelline (N'-methylnicotinate)	2.09	1.93	1.63	0.021	0.008	0.018
Xenobiotics								
	Bacterial/Fungal	N-methylpipecolate	1.53	1.40	1.85	0.038	0.042	0.037

Note: Fold change is relative to CON-CON at day 130 gestation in each tissue. Calculated relative fold change values were compared to 1 using t-tests. Significance denoted at $P \leq 0.05$.
Abbreviations: RES-CON = restricted day 50 to 90, control day 90 to 130; CON-CON = control day 50 to 130 gestation.

Table S5: Differential metabolites common across liver, muscle, and blood in RES-RES at day 130 of gestation.

			Fold Change			*P*-value		
Super Pathway	Sub Pathways	Metabolites	Liver	Muscle	Blood	Liver	Muscle	Blood
Amino Acid/Peptide								
	Histidine Metabolism	1-methylhistidine	1.40	1.40	1.52	0.007	0.020	0.020
	Lysine Metabolism	2-aminoadipate	2.38	1.53	1.61	<0.001	0.020	0.002
	Glutathione Metabolism	2-hydroxybutyrate/2-hydroxyisobutyrate	1.24	1.66	1.52	0.046	0.005	0.005
	Tyrosine Metabolism	3-(4-hydroxyphenyl)lactate	-1.19	-1.18	-1.37	0.031	0.027	0.001
	Glycine, Serine and Threonine Metabolism	betaine	-1.32	-1.28	-1.20	0.010	0.006	0.028
	Glycine, Serine and Threonine Metabolism	dimethylglycine	-1.67	-1.64	-1.67	0.002	<0.001	0.002
	Gamma-glutamyl Amino Acid	gamma-glutamylthreonine	-1.28	-1.69	-1.56	0.033	0.001	0.001
	Histidine Metabolism	imidazole lactate	-1.41	-1.32	-1.41	0.007	0.011	0.002
	Tryptophan Metabolism	indolelactate	-1.69	-1.39	-1.61	0.002	0.002	<0.001
	Urea cycle; Arginine and Proline Metabolism	N-delta-acetylornithine	-1.30	-1.39	-1.33	0.021	0.007	0.039
	Urea cycle; Arginine and Proline Metabolism	N-methylproline	-1.64	-1.35	-1.47	<0.001	0.014	0.007
	Phenylalanine Metabolism	phenyllactate (PLA)	-1.37	-1.20	-1.37	0.002	0.022	0.002
	Glycine, Serine and Threonine Metabolism	threonine	-1.27	-1.30	-1.25	0.004	0.002	0.008
	Urea cycle; Arginine and Proline Metabolism	urea	1.29	1.34	1.32	0.005	0.004	0.003

Carbohydrate								
	Fructose, Mannose and Galactose Metabolism	fructose	-1.45	-1.23	-1.41	<0.001	0.003	<0.001
	Advanced Glycation End-product	N6-carboxymethyllysine	-1.23	-1.39	-1.39	0.013	<0.001	<0.001
Lipid								
	Fatty Acid, Amino	2-aminoheptanoate	-1.67	-1.64	-1.72	<0.001	<0.001	<0.001
	Fatty Acid, Dicarboxylate	2-hydroxyadipate	1.53	1.83	1.50	0.004	0.002	0.029
	Primary Bile Acid Metabolism	cholate	-4.76	-7.14	-11.11	<0.001	<0.001	<0.001
	Phospholipid Metabolism	choline phosphate	-1.37	-1.10	-1.35	0.017	0.006	0.002
	Primary Bile Acid Metabolism	glycocholate	-1.89	-3.13	-2.44	<0.001	<0.001	<0.001
	Secondary Bile Acid Metabolism	glycodeoxycholate	-3.70	-2.33	-7.69	<0.001	0.011	<0.001
	Fatty Acid, Dicarboxylate	octadecanedioate (C18-DC)	1.32	1.33	1.29	0.037	0.017	0.039
Nucleotide								
	Purine Metabolism, Guanine containing	7-methylguanine	-1.14	-1.14	-1.47	0.035	0.005	0.011
Xenobiotics								
	Drug - Topical Agents	2,6-dihydroxybenzoic acid	-1.96	-1.37	-1.64	<0.001	0.01	0.005
	Benzoate Metabolism	4-ethylphenylsulfate	-1.72	-1.32	-1.64	<0.001	0.006	<0.001
	Benzoate Metabolism	4-methylcatechol sulfate	-1.92	-1.56	-1.72	0.005	0.015	0.014
	Benzoate Metabolism	catechol sulfate	-1.52	-1.45	-1.64	0.026	0.024	<0.001
	Food Component	mannonate	-1.39	-1.16	-1.25	0.011	0.012	0.039

Note: Fold change is relative to CON-CON at day 130 gestation in each tissue. Calculated relative fold change values were compared to 1 using t-tests. Significance denoted at $P \leq 0.05$.

Abbreviations: RES-RES = restricted day 50 to 90, restricted day 90 to 130 gestation; CON-CON = control day 50 to 130 gestation

Table S6: Differential Metabolites common across liver, muscle, and blood in RES-CON relative to RES-RES at day 130 of gestation.

			Fold Change			*P*-value		
Super Pathway	Sub Pathway	Biochemical Name	Liver	Muscle	Blood	Liver	Muscle	Blood
Amino Acid/Peptide								
	Histidine Metabolism	1-methylhistidine	-1.85	-1.65	-2.00	<0.001	<0.001	<0.001
	Lysine Metabolism	2-aminoadipate	-1.89	-1.28	-1.63	0.001	0.017	<0.001
	Glutathione Metabolism	2-hydroxybutyrate/2-hydroxyisobutyrate	-1.31	-1.75	-1.(67	0.003	<0.001	<0.001
	Histidine Metabolism	3-methylhistidine	-1.60	-1.26	-1.67	<0.001	0.005	0.018
	Glutamate Metabolism	4-hydroxyglutamate	-1.29	-1.14	-1.20	0.003	0.029	0.001
	Histidine Metabolism	imidazole propionate	-2.26	-1.92	-1.87	<0.001	0.003	0.002
	Lysine Metabolism	N6,N6-dimethyllysine	-1.39	-1.18	-1.15	0.002	0.042	0.003
	Glycine, Serine and Threonine Metabolism	N-acetylglycine	-1.34	-1.28	-1.37	<0.001	0.018	0.008

	Lysine Metabolism	pipecolate	1.27	1.60	1.28	0.018	0.006	0.034
	Urea cycle; Arginine and Proline Metabolism	urea	-1.27	-1.25	-1.33	0.001	0.001	<0.001
Carbohydrate								
	Aminosugar Metabolism	erythronate	1.55	1.38	1.31	0.008	0.007	0.014
	Fructose, Mannose and Galactose Metabolism	fructose	1.31	1.36	1.37	0.003	0.006	<0.001
	Advanced Glycation End-product	N6-carboxymethyllysine	1.15	1.20	1.25	0.042	<0.001	<0.001
Lipid								
	Fatty Acid, Amino	2-aminoheptanoate	1.49	1.74	1.72	<0.001	<0.001	<0.001
	Fatty Acid, Dicarboxylate	2-hydroxyadipate	-1.43	-1.50	-1.47	0.017	0.002	<0.001
	Ketone Bodies	3-hydroxybutyrate (BHBA)	-1.31	-1.94	-2.06	0.017	<0.001	<0.001
	Fatty Acid, Dicarboxylate	octadecenedioate (C18:1-DC)*	-1.35	-1.31	-1.33	0.016	0.008	0.003

	Secondary Bile Acid Metabolism	taurohyodeoxycholic acid	-1.22	-2.68	-1.95	0.041	<0.001	0.003
	Phospholipid Metabolism	trimethylamine N-oxide	1.57	1.58	1.79	0.001	<0.001	0.023
Xenobiotics								
	Benzoate Metabolism	2-hydroxyhippurate (salicylurate)	2.14	1.69	1.64	0.014	0.026	0.026
	Benzoate Metabolism	4-ethylphenylsulfate	1.67	1.46	1.57	0.006	0.013	<0.001
	Benzoate Metabolism	4-methylcatechol sulfate	1.76	1.55	1.51	0.012	0.014	0.010
	Benzoate Metabolism	hippurate	1.68	1.54	1.47	0.005	0.015	0.005

Note: Fold change is relative to RES-RES at day 130 gestation in each tissue. Calculated relative fold change values were compared to 1 using Student's t-tests. Significance denoted at $P \leq 0.05$.
Abbreviations: RES-CON = restricted day 50 to 90, control day 90 to 130; RES-RES = restricted day 50 to 130 gestation.
